# Co-development of a contextually relevant maternity service user feedback tool in Malawi using participatory consensus methods

**DOI:** 10.1186/s12913-026-15201-x

**Published:** 2026-07-24

**Authors:** Mtisunge Joshua Gondwe, Yamikani Chimwaza, Chifundo Kondoni, Leonard Mndala, Annie Kuyere Khumalo, Nyadani Chitsulo, Bertha Maseko, Catherine Bamuya, Luis Gadama, Lily Kumbani, Effie Chipeta, Elizabeth Chodzaza, David Lissauer, Alinane Linda Nyondo-Mipando

**Affiliations:** 1 Maternal and Fetal Group, Liverpool Wellcome Programme, Blantyre, Malawi; 2 Malawi Epidemiology and Intervention Research Unit, Lilongwe, Malawi; 3 Department of Women’s and Children’s Health, University of Liverpool, Liverpool, UK; 4 Department of Obstetrics and Gynaecology, Kamuzu University of Health Sciences, Blantyre, Malawi; 5 Department of Midwifery, School of Maternal and Reproductive Health, Kamuzu University of Health Sciences, Blantyre, Malawi; 6 Centre for Reproductive Health, Kamuzu University of Health Sciences, Blantyre, Malawi; 7 Department of Health Systems and Policy, Kamuzu University of Health Sciences, Blantyre, Malawi

**Keywords:** Maternity care, Patient-reported experience measures, Co-design, Quality improvement, Participatory research, Malawi

## Abstract

**Background:**

Improving the quality of maternity care requires systematic incorporation of women’s experiences into quality improvement processes. Although several instruments exist to measure maternity care experiences, few have been developed for routine feedback within low-resource health systems, and existing tools used in Malawi are generic and do not adequately capture maternity-specific experiences. This study aimed to co-develop and establish stakeholder-informed content validity for a context-specific maternity service user feedback tool for routine quality improvement in Malawi.

**Methods:**

We conducted a qualitative participatory consensus study involving two multi-stakeholder workshops held in Malawi between July 2024 and February 2025. Stakeholders included maternity service users, healthcare providers, policymakers, programme implementers, researchers, and community representatives. The tool was developed through a structured pathway comprising preparatory stakeholder engagement, co-design, prototype development, iterative refinement, and validation. World Café discussions, group discussions, gallery walks, structured voting, and thematic analysis were used to identify, prioritise, and refine feedback domains, indicators, and delivery modalities.

**Results:**

A total of 48 stakeholders participated in the co-design workshop and 39 participated in the validation workshop. Through iterative stakeholder consensus, approximately 300 recommendations were synthesised into 35 descriptive categories and subsequently refined into seven priority domains: respectful and dignified care; communication and health education; emotional support; timely and equitable access; continuity and coordination of care; quality of services received; and overall satisfaction. These domains informed the development of a maternity service user feedback tool comprising 27 antenatal care, 28 labour and delivery, and 32 postnatal care indicators organised into three modular questionnaires. Stakeholders recommended piloting both paper-based and electronic versions to support feasible implementation within routine maternity quality improvement systems.

**Conclusion:**

This study developed a stakeholder-informed maternity service user feedback tool tailored to the Malawian health system. The findings establish the tool’s contextual relevance and stakeholder-informed content validity while providing a foundation for future evaluation of feasibility, reliability, construct validity, and implementation within routine maternity quality improvement programmes.

**Supplementary Information:**

The online version contains supplementary material available at https://doi.org/10.1186/s12913-026-15201-x.

## Background

Despite substantial improvements in maternity service utilisation globally, maternal mortality remains unacceptably high in many low- and middle-income countries (LMICs) [1]. In 2020, an estimated 287,000 women died from pregnancy-related causes, with approximately 70% of deaths occurring in sub-Saharan Africa [1]. Although more than 95% of pregnant women in Malawi attend at least one antenatal care (ANC) visit and deliver in a health facility, the maternal mortality ratio remains high, indicating persistent gaps in the quality and experience of maternity care rather than access alone [2].

Several validated instruments have been developed to assess women’s experiences of maternity care, including the Childbirth Experience Questionnaire, Pregnancy and Maternity Care Patients’ Experiences Questionnaire, Person-Centred Maternity Care Scale, Mothers on Respect Index, and Patient Satisfaction Questionnaire [3–7]. These instruments measure important dimensions such as respectful care, communication, decision-making, emotional support, and satisfaction. However, most were developed primarily for research or evaluation purposes rather than routine quality improvement within national health systems. Furthermore, many focus on specific phases of maternity care or individual dimensions of experience, limiting their usefulness as integrated service feedback mechanisms across the continuum of antenatal, intrapartum, and postnatal care.

In Malawi, the Ministry of Health has introduced generic client feedback mechanisms through the Quality Management Directorate to support monitoring of health service quality. These tools are typically administered through face-to-face interviews and assess broad aspects of patient satisfaction across different health services [7–9]. While useful for general quality assurance, they are not tailored to maternity care and therefore provide limited information on women’s experiences of respectful maternity care, communication, informed decision-making, continuity of care, and other issues specific to pregnancy, childbirth, and the postnatal period. Consequently, opportunities to identify maternity-specific quality gaps and guide targeted service improvement remain limited.

Unlike existing maternity experience instruments, the proposed feedback tool was designed specifically for routine use within Malawi’s health system through a participatory co-development process involving service users, healthcare providers, policymakers, programme implementers, and community representatives. Rather than measuring selected aspects of maternity experience alone, the tool integrates multiple domains identified by stakeholders across the continuum of maternity care and was designed to support continuous quality improvement, accountability, and routine service monitoring within resource-constrained settings.

The primary objective of this study was to co-develop a context-specific maternity service user feedback tool through a participatory consensus process involving key stakeholders in Malawi. Secondary objectives were to establish stakeholder-informed content relevance and contextual acceptability, refine prototype delivery modalities, and prepare the tool for subsequent pilot implementation and psychometric evaluation.

## Methods

### Study design and development process

This study employed a qualitative participatory consensus design to co-develop a context-specific maternity service user feedback tool for routine quality improvement in Malawi. The study was informed by principles of participatory action research [10], experience-based co-design [11], and the WHO framework for quality maternal and newborn care [12], which emphasize meaningful stakeholder engagement in intervention and tool development.

The development process followed five sequential phases: (1) preparatory stakeholder engagement and review of existing feedback mechanisms; (2) participatory co-design of tool content and delivery modalities; (3) prototype development and iterative refinement; (4) stakeholder validation of content, usability, and contextual relevance; and (5) preparation for pilot implementation. The present paper reports the first four phases. Psychometric evaluation, implementation feasibility, and routine use of the tool will be reported separately. The study is reported in accordance with the Consolidated Criteria for Reporting Qualitative Research (COREQ) [13].

### Study setting

The study was conducted in Malawi between July 2024 and April 2025. Two multi-stakeholder consensus workshops were held in Dowa District (co-design workshop) and Mangochi District (validation workshop). Workshop venues were purposively selected to provide a neutral environment conducive to extended participatory discussions and collaborative decision-making. Prototype refinement and stakeholder consultations were subsequently undertaken remotely and through follow-up meetings between September 2024 and April 2025.

### Stakeholder identification and participant selection

Participants were identified through a structured stakeholder mapping exercise conducted during the preparatory phase. The mapping aimed to ensure representation from all groups involved in the planning, delivery, governance, evaluation, and use of maternity services in Malawi.

Eligible participants were individuals who met at least one of the following criteria: were directly involved in planning, delivering, supervising, regulating, or evaluating maternity services; represented organisations responsible for maternal and newborn health policy or programme implementation; were recognised experts in maternal health, quality improvement, health systems, or implementation research; represented community organisations, disability organisations, or women’s groups; or had recent lived experience of receiving maternity services. Purposive sampling was used to recruit participants with expertise relevant to maternity care and health system implementation. Snowball sampling was subsequently employed whereby invited stakeholders recommended additional individuals with complementary expertise or community representation. Several participants were identified through existing professional collaborations with the research team to ensure inclusion of key national stakeholders responsible for maternal health policy and service delivery. Participants were recruited from the Ministry of Health, district health offices, healthcare facilities, professional associations, academic institutions, research organisations, development partners, non-governmental organisations, disability advocacy organisations, community representatives, and maternity service users. This broad stakeholder representation was intended to maximise diversity of perspectives and improve the relevance, acceptability, and feasibility of the final feedback tool.

### Facilitator training

Seven facilitators (two male and five female) received structured training before each workshop under the supervision of an experienced qualitative researcher (ALNM). Training covered participatory facilitation techniques, management of group dynamics, use of discussion guides, reflexivity, documentation procedures, and strategies to promote equitable participation. Particular emphasis was placed on encouraging contributions from community women and other stakeholders who might otherwise be less likely to participate in mixed professional groups.

### Participatory workshops

The co-design and validation workshops followed a common structure comprising plenary presentations, facilitated small-group discussions, World Café sessions, gallery walks, structured voting exercises (co-design workshop only), and plenary consensus discussions. These activities were designed to progressively generate, refine, prioritise, and validate feedback domains, questionnaire items, and preferred delivery modalities.

### Participatory techniques

#### World café

Participants rotated between discussion stations reviewing and refining outputs generated by preceding groups. At each station, they could modify, expand, merge, or prioritise proposed indicators until consensus emerged regarding the content of the feedback tool.

#### Gallery walk

During gallery walks, draft questionnaires and prototype delivery modalities were displayed around the room. Participants independently reviewed materials, recorded written comments on sticky notes, and discussed suggested modifications with other participants. Feedback was subsequently synthesised during plenary sessions and incorporated into iterative revisions.

#### Consensus process

Although electronic administration received the highest number of votes during structured voting, subsequent plenary discussions identified important implementation considerations including variable internet connectivity, electricity supply, digital literacy, and facility resources. Stakeholders therefore reached consensus that both paper-based and electronic versions should be piloted to maximise accessibility while allowing comparison of feasibility, usability, and data management under routine conditions.

### Data analysis

Data comprised audio-recorded discussions, facilitator notes, flip charts, gallery walk comments, voting outputs, photographs of workshop materials, and field observations. Analysis followed the six stages of reflexive thematic analysis. First, all transcripts and workshop materials were reviewed repeatedly to achieve familiarisation with the data. Second, each facilitator independently generated initial codes from discussions conducted within their assigned group. Codes represented stakeholder priorities, concerns, proposed indicators, implementation considerations, and suggested revisions. Third, similar codes were grouped into descriptive categories through iterative cross-group comparisons. Fourth, categories were synthesised into candidate themes representing broader domains of maternity care experiences. Fifth, all seven analysts met during structured consensus meetings to compare coding outputs, discuss divergent interpretations, merge overlapping categories, and agree on final themes. Disagreements were resolved through discussion until consensus was reached. Finally, themes were reviewed against the original data to ensure they accurately reflected stakeholder perspectives before informing revisions to the feedback tool. Manual coding was selected because the dataset was generated through a limited number of intensive consensus workshops that incorporated multiple forms of qualitative evidence, including transcripts, visual materials, voting outputs, and field observations. Manual analysis enabled simultaneous interpretation of textual and visual data while maintaining close engagement with stakeholder discussions. An audit trail documenting coding decisions, theme development, and successive revisions was maintained throughout analysis to enhance dependability and transparency.

### Reflexivity

The multidisciplinary analytical team included researchers with backgrounds in obstetrics, midwifery, nursing, implementation science, qualitative research, and health systems. Several analysts had existing professional relationships with participating organisations. To minimise potential bias, coding decisions were documented throughout analysis, alternative interpretations were actively discussed during team meetings, and final themes were agreed through consensus rather than individual interpretation.

### Translation and linguistic validation

Questionnaires were initially developed in English before being translated into Chichewa by bilingual maternal health professionals. The translated versions were independently reviewed by additional bilingual experts to ensure conceptual rather than literal equivalence. Discrepancies were reconciled through consensus discussions involving translators and members of the research team. During the validation workshop, participants assessed both language versions for clarity, cultural appropriateness, and comprehensibility, leading to further refinements before finalisation.

### Confidentiality

During workshop activities, stakeholders were encouraged to discuss sensitive topics, including mistreatment, informal payments, and complaints. All discussions were conducted in private meeting venues, audio recordings were anonymised during transcription, and quotations were de-identified before reporting. The final tool was designed to allow anonymous completion by maternity service users to minimise fear of reprisal and encourage honest feedback during future implementation.

### Ethical approval

Ethical approval for this study was obtained from the College of Medicine Research Ethics Committee (COMREC; P.07/23–0167) in Malawi and the University of Liverpool Research Ethics Committee (UoL13920). The study was conducted in accordance with the ethical principles of the Declaration of Helsinki and relevant institutional guidelines. Participation was entirely voluntary, and written informed consent was obtained from all participants before data collection. Participants were informed of their right to decline participation or withdraw from the study at any time without consequence. To facilitate participation, transport, accommodation, and subsistence costs were reimbursed in accordance with institutional ethical guidance; these reimbursements were not considered payment for participation. Participant anonymity and confidentiality were maintained throughout the study by assigning unique identifiers, removing personal identifiers from transcripts and study records, and securely storing all data in password-protected systems accessible only to authorised members of the research team.

## Results

### Participant characteristics

A total of 48 stakeholders participated in the co-design workshop and 39 participated in the validation workshop, representing government departments, healthcare providers, researchers, academic institutions, professional associations, development partners, community representatives, organisations of persons with disabilities, and civil society organisations (Table 1). Approximately half of the validation participants had also participated in the co-design workshop, ensuring continuity throughout the development process while incorporating independent perspectives from newly engaged stakeholders. Stakeholder participation reflected broad representation from organisations involved in maternity service delivery, governance, education, research, community engagement, and quality improvement. Structured facilitation techniques, including purposive group composition, rotating facilitators, and guided discussion questions, promoted equitable participation and ensured that community representatives and women with lived maternity care experience contributed meaningfully to questionnaire wording, respectful maternity care indicators, accessibility, communication, and overall usability of the tool.

**Table 1:** Characteristics of stakeholders participating in the co-design and validation workshops

Characteristic	Co-design Workshop (*n* = 48)	Validation Workshop (*n* = 39)
Government and Ministry of Health representatives	7	10
Healthcare providers and facility managers*	19	10
Training and academic institutions	3	4
Professional associations	–	1
Research institutions	10	7
Community representatives (women/service users)	5	5
Organisations representing persons with disabilities	1	2
Local NGOs	1	–
Development partners/donor organisations	1	–
**Total participants**	**48**	**39**

*Healthcare providers and facility managers included obstetricians, clinical officers, nurse/midwives, district medical officers, district nursing and midwifery officers, safe motherhood coordinators, and hospital ombudsmen

### Stakeholder-derived domains of maternity care experience

Analysis of data generated during the co-design workshop, including transcripts, facilitator notes, flip charts, gallery walk comments, and stakeholder voting outputs, produced approximately 300 stakeholder recommendations relating to women’s experiences across antenatal, labour and delivery, and postnatal care. Through iterative coding and thematic analysis, these recommendations were progressively synthesised into broader conceptual categories and subsequently grouped into seven overarching domains representing the dimensions of maternity care considered most important by stakeholders (Supplementary Table 1).

The seven domains comprised respectful and dignified care; communication and health education; emotional support; timely and equitable access; continuity and coordination of care; quality of services received; and overall satisfaction. Stakeholder priorities were remarkably consistent across professional and community groups, with no major disagreement regarding the seven final domains. Together, these domains formed the conceptual framework underpinning the maternity-specific feedback tool and demonstrated close alignment with the WHO framework for quality maternal and newborn care (Table 2).

**Table 2:** Stakeholder-derived domains, corresponding WHO Quality of Care domains, and representative questionnaire indicators

Stakeholder-Derived Theme	Summary of Stakeholder Priorities	Corresponding WHO Quality of Care Domain	Representative Questionnaire Indicators
Respectful and Dignified Care	Women should be treated with dignity, respect, privacy, and compassion throughout maternity care. Participants also emphasised informed consent, equitable treatment, inclusion of women with disabilities, and the opportunity to have a birth companion.	Experience of Care – Respect and Preservation of Dignity; Effective Communication; Emotional Support	Privacy maintained during consultations and examinations; respectful provider interactions; informed consent obtained; birth companion permitted where appropriate; respectful care for women with disabilities.
Communication and Health Education	Women should receive clear, understandable information about their care, clinical procedures, investigations, treatment, newborn care, and follow-up recommendations to support informed decision-making.	Experience of Care – Effective Communication	Healthcare providers explained procedures clearly; opportunities to ask questions; understanding of care plan; health education received; discharge and follow-up instructions understood.
Emotional Support and Companionship	Emotional reassurance, encouragement, psychosocial support, companionship during labour, and appropriate bereavement support were considered important components of quality maternity care.	Experience of Care – Emotional Support	Companion present during labour (where permitted); emotional support provided; counselling following pregnancy or newborn loss; referral for psychosocial support where needed.
Timely and Equitable Access to Care	Stakeholders prioritised prompt access to services, reasonable waiting times, equitable treatment, accessibility for women with disabilities, and adequate facility infrastructure.	Cross-cutting WHO Domain – Availability of Physical Resources and Experience of Care	Waiting time before receiving care; accessibility of facilities; availability of essential services; cleanliness and comfort of the facility; equitable access irrespective of disability or social status.
Continuity and Coordination of Care	Women should experience coordinated care across antenatal, labour and delivery, and postnatal services, including appropriate referrals, follow-up, and continuity of information.	Provision of Care – Appropriate Referral Systems; Actionable Information Systems	Follow-up appointments arranged; referrals completed; continuity across maternity services; information transferred between providers; visibility of complaints and ombudsman services.
Quality of Clinical Services Received	Participants emphasised receiving recommended clinical assessments, medicines, laboratory investigations, preventive interventions, and newborn care without informal payments.	Provision of Care – Evidence-Based Clinical Practice; Availability of Essential Physical Resources	Recommended ANC, labour and postnatal services received; essential medicines and supplies available; laboratory investigations completed; no informal payments requested; quality of newborn care.
Overall Satisfaction with Maternity Care	Overall perceptions of care should capture women’s experiences across all stages of maternity care while identifying priorities for service improvement.	Experience of Care – Person-Centred Care	Overall satisfaction rating; expectations met; willingness to recommend the facility; suggestions for improving maternity services.

Respectful and dignified care emerged consistently across all stakeholder groups as the most important dimension of women’s maternity care experiences. Stakeholders highlighted respectful communication, preservation of privacy, informed consent, equitable treatment, provider attitudes, and the opportunity for women to be accompanied by a support person as essential components of high-quality maternity care. Communication and health education also emerged as a major priority. Stakeholders emphasised that women should receive clear explanations regarding clinical procedures, labour progress, newborn care, and follow-up recommendations throughout the maternity care continuum. Effective communication was viewed as essential for informed decision-making, reducing anxiety, and strengthening trust between women and healthcare providers. Emotional support was identified as another key component of quality maternity care. Participants highlighted the importance of reassurance, encouragement, psychosocial support during labour, and appropriate bereavement support for women experiencing pregnancy or newborn loss. Stakeholders also prioritised timely and equitable access to maternity services, emphasising waiting times, equitable treatment regardless of personal circumstances, and the availability of essential services throughout pregnancy, childbirth, and the postnatal period. Continuity and coordination of care were considered important for ensuring effective follow-up, referral pathways, continuity across different stages of maternity care, and consistent communication between healthcare providers. Finally, stakeholders recommended incorporating indicators relating to the quality of services received, including availability of essential medicines and supplies, as well as overall satisfaction with maternity care to support routine monitoring and continuous quality improvement.

### Development and refinement of questionnaire items

Approximately 300 stakeholder-generated recommendations were consolidated into 35 descriptive categories (Supplementary Table 1), synthesised into seven stakeholder-derived domains (Supplementary Fig. 1), and subsequently translated into three modular questionnaires. The analytical pathway from stakeholder-generated recommendations to the final questionnaire is illustrated in Supplementary Fig. 1. Throughout the iterative development process, duplicate indicators were merged, ambiguous wording was revised, and items considered impractical for routine implementation were removed following stakeholder consensus. Consensus was considered achieved when no substantive objections remained following plenary discussion. This refinement process prioritised conceptual relevance, contextual appropriateness, and implementation feasibility while maintaining comprehensive coverage of stakeholder priorities. The resulting maternity-specific feedback tool comprised 27 antenatal care indicators, 28 labour and delivery indicators, and 32 postnatal care indicators organised into three modular questionnaires. The progression from stakeholder recommendations to the final questionnaire structure is summarised in Supplementary Table 2.

### Accessibility and disability-inclusive adaptations

Stakeholders representing organisations of persons with disabilities contributed several recommendations to improve the accessibility and inclusiveness of the feedback tool. These recommendations included expanding demographic questions to capture disability status, simplifying question wording, using respectful terminology, improving readability, and ensuring that women requiring assistance could complete the questionnaire while maintaining confidentiality. Participants also recommended maintaining both paper-based and electronic administration options to accommodate different facility contexts and varying user needs. These recommendations informed revisions to questionnaire wording, demographic sections, administration guidance, and implementation procedures incorporated into the final tool.

### Selection of tool delivery modalities

Stakeholders evaluated five potential delivery modalities using structured group discussions, gallery walks, and voting (Table 3). Electronic questionnaires received the highest number of votes because of their perceived advantages for real-time data capture, automated reporting, and improved data management. Paper-based questionnaires were considered inexpensive, familiar to healthcare workers, and suitable for facilities with limited digital infrastructure, whereas mobile applications and ACASI offered additional flexibility but required greater technological investment. Across all modalities, stakeholders emphasised the importance of staff training, community sensitisation, government ownership, supervision, and integration into existing quality improvement systems. Although electronic administration was initially preferred, plenary discussions highlighted practical implementation challenges, including unreliable electricity supply, intermittent internet connectivity, equipment costs, maintenance requirements, and varying levels of digital literacy. Stakeholders therefore reached consensus that both paper-based and electronic versions should be piloted before selecting a preferred implementation strategy.

**Table 3:** Stakeholder preferences for feedback tool delivery modalities and decision following stakeholder consensus

Tool Delivery Modality	Number of Votes (*n* = 51)	Perceived Advantages Identified by Stakeholders	Implementation Considerations	Decision following stakeholder consensus
Electronic questionnaire	16	Real-time data capture, automated reporting, reduced data entry errors, improved data management	Dependent on electricity, internet connectivity, digital literacy, device availability, and ongoing technical support	Selected for pilot testing
Paper-based questionnaire	13	Simple to administer, familiar to healthcare workers, suitable for facilities with limited digital infrastructure	Requires manual data entry, increased risk of transcription errors, slower reporting	Selected for pilot testing
Mobile application	12	Potential for community-based data collection and rapid reporting	Requires smartphone access, software development, user training, and maintenance	Not selected for initial pilot
Audio computer-assisted self-interview (ACASI)	8	May improve privacy, confidentiality, and accessibility for respondents with low literacy	Requires specialised equipment, software, headphones, and technical support	Not selected for initial pilot
Combined approach	2	Flexibility across different contexts	Operational complexity and higher implementation costs	Considered during discussions but not prioritised

**Table 4:** Structure of the final maternity service user feedback tool and key revisions following stakeholder validation

Module	No. of Items	Purpose and scope	Key content areas	Major revisions following stakeholder validation
Antenatal Care (ANC) Feedback Form	24	Evaluates women’s experiences during initial and follow-up antenatal care visits, with emphasis on respectful, person-centred care and service accessibility.	Facility characteristics; reception and orientation; clinical assessment and investigations; medicines and preventive interventions; health education; waiting time; respectful care; communication; informal payments; accessibility; disability inclusion; expectations; overall satisfaction.	Simplified medical terminology; revised questionnaire title; expanded demographic section (including education and disability status); improved wording of questions on provider rapport, respectful care, abuse, communication, expectations, and satisfaction; repositioned disability-related items for better flow; added prompts to capture details of informal payments.
Labour and Delivery Feedback Form	24	Assesses women’s experiences during admission, labour, childbirth, and immediate postpartum care, with particular emphasis on informed consent, respectful care, and newborn care.	Admission and ward orientation; labour and delivery processes; informed consent; provider identification; maternal and newborn care; respectful care; communication; companionship; informal payments; disability inclusion; accessibility; satisfaction.	Incorporated revisions applied to the ANC module; refined questions relating to vaginal examinations, labour progress, contractions, and birth companionship; added information on baby’s sex, condition at birth, and time of delivery; improved wording of questions relating to informal payments and overall satisfaction.
Postnatal Care (PNC) Feedback Form	25	Evaluates women’s experiences across the WHO-recommended postnatal care schedule, focusing on continuity of care, maternal and newborn outcomes, and ongoing support.	Ward orientation; maternal and newborn services at 48 h, 7 days and 6 weeks; communication; respectful care; waiting time; accessibility; disability inclusion; informal payments; continuity of care; satisfaction; recommendations for improvement.	Incorporated revisions made to ANC and labour modules; added questions on date and mode of delivery, separate care received by mother and newborn, nursery admission, HIV exposure, kangaroo mother care, and psychological support following infant loss; standardised wording, response options, and payment-related questions across all modules.

The number of questionnaire items differs from the number of indicators because several items capture multiple quality indicators or include branching questions

### Findings from the validation workshop

The validation workshop focused on assessing the relevance, clarity, usability, accessibility, and contextual appropriateness of the draft feedback tool. Participants reviewed both English and Chichewa versions of the paper-based and electronic questionnaires through facilitated group discussions, practical testing sessions, gallery walks, and plenary consensus discussions. Validation produced refinements across every section of the questionnaires (Supplementary Table 2). For example, during validation, “Were you treated well?” was revised to “Were you treated respectfully?” to improve clarity and consistency with respectful maternity care terminology. Common changes across all three modules included simplification of medical terminology, standardisation of response options, revision of respectful maternity care questions, improved wording relating to communication and provider interaction, expansion of demographic information to include education and disability status, repositioning of disability-related items, and clarification of payment-related questions. Module-specific revisions included the addition of infant condition and time of delivery in the labour questionnaire, and questions relating to newborn admission, kangaroo mother care, HIV exposure, and bereavement support in the postnatal questionnaire. These revisions improved clarity, contextual relevance, and completeness before prototype finalisation. These revisions were subsequently incorporated through two additional rounds of stakeholder review before finalisation of the prototype.

### Feasibility and respondent burden

Throughout the validation process, stakeholders considered the balance between comprehensive measurement and practical implementation. Rather than developing a single lengthy questionnaire, participants supported organising the tool into three separate modules corresponding to antenatal care, labour and delivery, and postnatal care. Stakeholders estimated that each module could be completed within approximately 10–20 min depending on care complexity and delivery modality. This modular approach was considered more feasible because women would complete only the questionnaire relevant to their stage of care, thereby reducing respondent burden while maintaining comprehensive assessment across the maternity care continuum. Participants further recommended that questionnaires be administered immediately following service provision or during scheduled follow-up visits in private settings to maximise recall while protecting confidentiality and encouraging honest feedback.

### Final maternity service user feedback tool

The final maternity-specific feedback tool (Table 4) comprised three modular questionnaires covering antenatal care (27 indicators), labour and delivery (28 indicators), and postnatal care (32 indicators). Across all three modules, indicators were organised into the seven stakeholder-derived domains of respectful and dignified care, communication and health education, emotional support, timely and equitable access, continuity and coordination of care, quality of services received, and overall satisfaction. The questionnaires were produced in both English and Chichewa and formatted for paper-based and electronic administration. The stakeholder-derived domains demonstrated strong conceptual alignment with the WHO Framework for Improving Quality of Maternal and Newborn Care while extending it through context-specific indicators relevant to the Malawian health system, including disability inclusion, informal payments, complaints mechanisms, and continuity across the maternity care pathway (Table 2). Stakeholders considered the final tool to be contextually relevant, acceptable, and suitable for pilot implementation. However, they recognised that formal psychometric evaluation, including assessment of reliability, construct validity, responsiveness, feasibility, and implementation performance, will be required before routine adoption within the national health system. The three-module structure enables stage-specific assessment across the maternity continuum while supporting routine quality improvement and accountability within health facilities.

## Discussion

This study co-developed a context-specific maternity service user feedback tool through a structured participatory consensus process involving maternity service users, healthcare providers, policymakers, researchers, programme implementers, and community representatives in Malawi. The resulting tool comprises three modular questionnaires covering antenatal, labour and delivery, and postnatal care, organised around seven stakeholder-derived domains reflecting women’s experiences across the continuum of maternity care. By combining participatory co-design, iterative refinement, and stakeholder validation, the study established the contextual relevance, acceptability, and stakeholder-supported content of the tool while preparing it for subsequent pilot testing and psychometric evaluation. Rather than validating measurement properties, this study focused on ensuring that the tool reflects the priorities, experiences, and implementation realities of those who will ultimately use it within routine maternity services.

### Participatory co-design strengthened contextual relevance and stakeholder ownership

The principal strength of this study lies in its participatory approach to tool development. Unlike many maternity experience instruments that are developed primarily by researchers or adapted from existing questionnaires, this tool was co-designed with stakeholders representing all major levels of the health system, including women with lived experience of maternity care, healthcare providers, health system managers, disability organisations, researchers, and policymakers. This approach ensured that the final instrument reflected both experiential and operational dimensions of maternity care while remaining feasible for routine implementation within the Malawian health system.

Participatory approaches are increasingly recognised as essential for developing health interventions that are contextually appropriate, acceptable to end users, and more likely to be adopted within routine practice [14–16]. Experience-based co-design and participatory action research emphasise that service users possess unique experiential knowledge that complements professional expertise and strengthens intervention design [14, 17]. In the present study, service users contributed directly to identifying priorities relating to respectful maternity care, communication, emotional support, accessibility, and overall acceptability, while healthcare providers and policymakers ensured that proposed indicators remained practical and aligned with existing service delivery processes. The combination of these perspectives enhanced both the relevance and implementability of the final tool.

The structured use of World Café discussions, gallery walks, small-group discussions, and iterative consensus-building also facilitated collective ownership of the tool. Rather than generating indicators independently, stakeholders progressively refined, prioritised, merged, and validated questionnaire items through multiple rounds of discussion and review. Such iterative consensus approaches have been shown to improve intervention acceptability and facilitate subsequent implementation within complex health systems [18–20].

### Extending existing maternity experience measurement tools

Several validated instruments exist to measure women’s experiences of maternity care, including the Person-Centred Maternity Care Scale, Mothers on Respect Index, Childbirth Experience Questionnaire, and Pregnancy and Maternity Care Patients’ Experiences Questionnaire [3, 7, 9]. These instruments have contributed substantially to understanding respectful maternity care and women’s experiences during pregnancy and childbirth. However, most were developed primarily for research, programme evaluation, or assessment of specific aspects of maternity care rather than routine quality improvement within national health systems.

The maternity service user feedback tool developed in this study differs in several important respects. First, it was designed explicitly for routine implementation within existing quality improvement systems rather than for one-time research assessments. Second, it covers the entire continuum of maternity care through separate antenatal, labour and delivery, and postnatal modules rather than focusing on a single episode of care. Third, stakeholder engagement identified several context-specific priorities, including disability inclusion, informal payments, visibility of complaints mechanisms, and continuity of care that are not routinely incorporated into existing maternity experience instruments but are highly relevant to service delivery in Malawi and similar low-resource settings. Finally, the development process itself ensured that questionnaire content reflected locally identified priorities while remaining aligned with internationally recognised quality-of-care frameworks.

### Alignment with the WHO quality of care framework

The seven stakeholder-derived domains closely correspond to the WHO Framework for Improving the Quality of Maternal and Newborn Care, particularly the domains relating to experience of care, provision of care, and availability of essential physical resources [12]. Respectful and dignified care, communication, emotional support, and overall satisfaction align directly with the WHO experience-of-care domains, while continuity of care, quality of clinical services, and timely access reflect key components of evidence-based clinical practice and health system responsiveness. Importantly, stakeholders also identified priorities extending beyond the WHO framework to address challenges encountered within the Malawian health system. These included indicators relating to informal payments, disability inclusion, functioning complaints mechanisms, and facility-level accessibility. Incorporating these context-specific issues demonstrates that global quality frameworks provide an important foundation for tool development but require adaptation to local implementation contexts to ensure relevance and responsiveness to service users’ experiences. Similar observations have been reported in other low- and middle-income countries where contextual adaptation has improved the implementation and utility of quality improvement interventions [14, 21, 22].

### Implications for routine quality improvement

Routine measurement of women’s experiences has considerable potential to strengthen accountability and support continuous quality improvement within maternity services. Although Malawi has introduced generic client feedback mechanisms through the Ministry of Health Quality Management Directorate, these tools are not designed specifically for maternity care and therefore capture limited information regarding respectful maternity care, communication, continuity of care, or other issues that women identified as priorities. The stakeholder-derived feedback tool developed in this study provides a structured mechanism for routinely capturing women’s experiences across the continuum of maternity care while generating actionable information for healthcare providers, facility managers, district health teams, and policymakers. The modular structure reduces respondent burden by allowing women to complete only the questionnaire relevant to their stage of care, while the availability of both paper-based and electronic versions accommodates varying levels of health system infrastructure. Participants further recommended integrating the tool within existing quality improvement processes, including routine facility review meetings, district quality improvement forums, and Ministry of Health reporting systems. Such integration could strengthen accountability by enabling facilities to identify service gaps, monitor changes over time, and evaluate the impact of quality improvement interventions. Successful implementation, however, will depend on more than routine data collection. Stakeholders emphasised the importance of establishing clear feedback loops in which findings are reviewed regularly, translated into facility-level action plans, monitored through existing quality improvement structures, and communicated back to service users where appropriate. Without systematic review and response mechanisms, the collection of user feedback alone is unlikely to improve the quality of maternity care.

### Strengths and limitations

This study has several strengths. The participatory design ensured representation from multiple stakeholder groups involved in maternity care planning, delivery, regulation, research, and service use. Multiple qualitative methods, including group discussions, World Café sessions, gallery walks, structured voting, and iterative validation, enabled triangulation of stakeholder perspectives and strengthened the credibility of the findings. The iterative development process further enhanced the contextual relevance, acceptability, and usability of the final tool. Several limitations should also be acknowledged. First, stakeholder recruitment relied primarily on purposive and snowball sampling, with some participants identified through existing professional networks. Although this approach ensured inclusion of key national stakeholders, it may have introduced selection bias by favouring individuals who were already engaged with maternal health programmes or supportive of quality improvement initiatives. Second, despite deliberate facilitation strategies designed to promote equitable participation, professional stakeholders may have influenced discussions more strongly than community representatives in some mixed groups. Although facilitators actively encouraged participation from women with lived experience and organisations representing persons with disabilities, it was not possible to eliminate all power dynamics inherent in consensus workshops. Third, approximately half of the validation participants had participated in the co-design workshop. While this continuity helped preserve institutional knowledge and ensured that earlier design decisions could be critically reviewed, it may also have increased agreement with previous recommendations. The inclusion of newly recruited participants partially mitigated this risk by providing independent perspectives during validation. Finally, this study established stakeholder-informed content relevance and contextual acceptability but did not evaluate the psychometric properties of the instrument. Reliability, construct validity, responsiveness, implementation feasibility, and routine operational performance remain to be established through pilot testing before wider implementation.

### Future research

The next phase of this work will involve pilot implementation within maternity facilities to assess feasibility, acceptability, questionnaire completion rates, administration time, and integration into routine health services. Subsequent psychometric evaluation will examine internal consistency, test–retest reliability, construct validity, criterion validity where appropriate, responsiveness to changes in service quality, and implementation outcomes. Successful implementation will depend not only on collecting feedback but also on organisational readiness, leadership engagement, supportive supervision, adequate resources, and mechanisms that translate feedback into measurable quality improvement actions. Future implementation research should therefore evaluate both implementation outcomes (acceptability, feasibility, fidelity, sustainability) and service outcomes. Future studies should also evaluate whether routine use of the feedback tool contributes to measurable improvements in respectful maternity care, service responsiveness, and maternal health outcomes. Cross-cultural adaptation and validation in other low-resource settings should also be explored.

## Conclusion

This study developed a stakeholder-informed maternity service user feedback tool that is contextually relevant to the Malawian health system and aligned with internationally recognised quality-of-care frameworks. Through participatory co-design and iterative stakeholder validation, the study established the content relevance, acceptability, and practical utility of the tool while incorporating locally identified priorities that extend existing maternity experience instruments. The resulting three-module questionnaire provides a structured approach for routinely capturing women’s experiences across antenatal, labour and delivery, and postnatal care. Although the tool is ready for pilot implementation, further evaluation is required to establish its psychometric properties, implementation feasibility, and effectiveness in supporting routine quality improvement and accountability within maternity services.

## Supplementary Information

Below is the link to the electronic supplementary material.

Supplementary Material 1: Figure S1: Development pathway of the maternity-specific feedback tool

Supplementary Material 2: Table S1: List of stakeholders invited and attended co-design and validation workshops

Supplementary Material 3: File S2: Questionnaire guide and participatory discussion guide

Supplementary Material 4: File S3: Detailed World Café Methods

Supplementary Material 5: File S4: Tool delivery modality assessment and consensus process

Supplementary Material 6: File S5: Stakeholder-generated questionnaire content from the co-design workshop.

Supplementary Material 7: Table S6: Stakeholder comparison of proposed tool delivery modalities.

Supplementary Material 8: Table S7: Stakeholder-recommended revisions following validation workshop

## Data Availability

The datasets generated and analysed during the current study are included in this published article and its supplementary information files. Additional de-identified data are available from the corresponding author upon reasonable request, subject to institutional ethical and data-sharing requirements.

## Declarations

### Patient and public involvement

Women with recent experience of maternity care and community representatives were actively involved throughout the study. They participated in stakeholder mapping, co-design workshops, validation workshops, and iterative refinement of the feedback tool. Their contributions informed the identification of priority domains, refinement of questionnaire items, improvement of language and accessibility, and selection of implementation modalities. Although patients and members of the public were not involved in determining the overall research questions or study design, they made substantial contributions to the development and contextualisation of the final maternity service user feedback tool.

## Abbreviations

ACASI: Audio Computer-Assisted Self-Interviewing
ANC: Antenatal Care
DHIS2: District Health Information Software 2
HMIS: Health Management Information System
LMICs: Low- and Middle-Income Countries
NGOs: Non-Governmental Organizations
PCMC: Person-Centred Maternity Care
PSQ-18: 18-item Patient Satisfaction Questionnaire
QMD: Quality Management Directorate
RHD: Reproductive Health Directorate
RMC: Respectful Maternity Care
