# Supplementary material for: Co-development of a contextually relevant maternity service user feedback tool in Malawi using participatory consensus methods
Source: BMC Health Serv Res. 2026 Jul 24;26:1444. doi: 10.1186/s12913-026-15201-x (PMC13689074; doi:10.1186/s12913-026-15201-x)
Supplement: Supplementary file 2 [file 12913_2026_15201_MOESM2_ESM.docx]

Supplementary File S4: Questionnaire guide and participatory discussion guide

**Day 1 –**

Organise participants into 5 or 6 tables – depending on numbers present, ensure mixed disciplines in each table.

Each table to appoint:

- a chairperson to facilitate and manage the discussion at the table
- a Scribe to take notes of the discussion at the table (Safe Motherhood staff can be here)
- a Rapporteur to feed back the discussion to the whole (plenary) group

**Discussion 1	Content of the tools – Indicators to capture (World Café)**

This should be initially done by each participant, writing the proposed content. These should then be placed on a flip chart under specific columns of:

- ANC,
- Labour and Delivery,
- PNC
- Other

The chair should then organise/order the post-its in discussion with the members – assess for completeness of ideas, including counting the numbers for each action.

- Health Centre Specific Tools
- District Level Tools
- Central Hospital Tools

Agree on the table’s list of content to be included and the rationales behind it. The Chair and Safe Motherhood Research staff remain at the table while the others visit other tables as a group. The Chair explains to the others who come, and they comment on the group’s work. Meanwhile, the Research Staff has a clean sheet that compiles everything for the group

**Discussion Two- Type/Format of Tools**

Gallery Walks – Let’s have stations that will have a type of tool:

Station 1- Paper-based tools

Station 2: Electronic-Based Tools

Station 3: Mobile App Tools

Station 4: Audio Tools/ACASI

Station 5: Combined Approaches

For stations 1-5, check the following:

Station Manager- Ask the groups to share/discuss the following

- Length of each tool
- Advantages
- Disadvantages
- Implementation of each tool
- Which facilities for which tool?
- Likelihood of implementing each tool (assess feasibility- resources needed, time to complete the tool)
  - Feasible – Can easily be administered once the tool is ready
  - Feasible with minimal support- need to change a few or improve things (state the areas needing support)
  - Feasible, but will require so much support (Describe the vast support)
  - Not feasible in certain facilities—state the facilities
  - Not feasible at all in public/CHAM facilities- specify the reasons

Station 6: Designing of the tool ( do for all tools):

- Who should be involved? Personnel, Organisation, who would need to be involved (at all levels- facility, PHC, District, Central, RHD, QMD, Donors)?
- What would be needed for it to be taken forward?
- Logistical Support
- Preliminary sites to pilot in
- What would facilitate Implementation
- What would hinder implementation
- Who can develop the tools? (specify for each tool)

**Priority Ranking**

Following the discussion, discuss and agree on a priority ranking for the type of tools and content

**Station 7: Implementation of the tool and Data from Feedback Mechanisms**

- Ethical considerations, Safeguarding policies, Anonymity measures
- Frequency
- When to administer
- Who will administer
- Languages and rationales
- Districts to pilot in—Interested partners to pilot with

**Data**

- Management of data
- Stakeholders, Patient Groups
- Security and confidentiality
- Dissemination and use of the data for research purposes
- Continued Collection

**Validation**

- Information and materials to be shared
- Stakeholders to Validate
- Possible dates
