# Supplementary material for: Co-development of a contextually relevant maternity service user feedback tool in Malawi using participatory consensus methods
Source: BMC Health Serv Res. 2026 Jul 24;26:1444. doi: 10.1186/s12913-026-15201-x (PMC13689074; doi:10.1186/s12913-026-15201-x)
Supplement: Supplementary file 3 [file 12913_2026_15201_MOESM3_ESM.docx]

**Supplementary File S5: Detailed World Café Methods**

**Phase one: Group discussion**

In the first phase of group discussion, all six groups focused on identifying the key types of information or indicators to be included in the maternity-specific feedback tool. Discussions were structured to address the entire continuum of care across general service quality, antenatal care, labour and delivery, and the postnatal period. In addition to identifying relevant indicators, participants were asked to determine the most appropriate level of the health system (primary, secondary, or tertiary) at which each type of information should be collected or applied. To support interactive and organized engagement, each group used adhesive sticky notes to record their suggested indicators, which were then displayed on flip charts. This method enabled participants to organize their ideas visually, facilitated collaborative prioritization, and allowed for cross-group sharing during plenary sessions. The visual format also supported transparency and traceability of the discussions, laying a foundation for consensus in subsequent phases.

**Phase two: Group discussions on delivery methods**

In the second phase of the group discussions, the first five groups (see Additional File 5) explored various modes of delivering the maternity-specific feedback tool, building on the insights gained in Phase 1. Each group was assigned a specific delivery format for in-depth examination: Group 1 focused on a paper-based version, Group 2 discussed an electronic-based version, Group 3 explored a mobile application, Group 4 examined an audio tool using Audio Computer-Assisted Self-Interviewing (ACASI), and Group 5 assessed the feasibility of a combined or hybrid approach. Each group discussed several key considerations, including the appropriate length of the tool, potential advantages and disadvantages of the proposed format, adaptability across different facility levels, and the overall feasibility in terms of required resources, time requirements, and ease of administration. Flip charts were used to visually organize and display group inputs, while voice recorders captured the full discussions for later analysis and review.

Group 6, composed of stakeholders with expertise in tool development, was tasked with conducting a critical evaluation of all five potential delivery methods. Their assessment focused on identifying the facilitators, barriers, and technical design requirements for each method. The inclusion of this expert group was instrumental in ensuring that the selected tool delivery approach would be contextually appropriate, user-friendly, and compatible with clinical practice. In addition to evaluating delivery formats, Group 6 also considered broader implementation issues (local workflows, resource availability, and staff capacity, enhancing its usability, adoption, and sustainability. These included stakeholder engagement, logistical requirements, pilot testing locations, ethical considerations, timing and frequency of tool administration, language and cultural appropriateness, data management, confidentiality, dissemination strategies, and mechanisms for routine data collection and analysis.

**World Café Sessions**

Following the initial group discussions on the content of the maternity-specific feedback tool and the appropriate levels of health system delivery, a World Café was employed to further support the participatory development and iterative refinement of the tool. This approach facilitated structured, small-group dialogue among a diverse mix of stakeholders as described in Additional file 5. Two rounds of 15-minute table discussions were conducted, with participants rotating between tables after each round. This rotation encouraged the cross-pollination of ideas and exposed participants to a broader range of perspectives. Each table was facilitated by a trained moderator who guided the discussions and summarized key points on flipcharts. Additionally, designated recorders captured verbatim comments to ensure a detailed and accurate account of stakeholder input. Following the World Café sessions, each group reconvened and presented its consolidated perspectives on what it considered to be the most appropriate content for inclusion in the maternity feedback tool. This collaborative and iterative approach helped to ensure that the tool reflected both consensus and diversity in stakeholder priorities.
