# Supplementary material for: Co-development of a contextually relevant maternity service user feedback tool in Malawi using participatory consensus methods
Source: BMC Health Serv Res. 2026 Jul 24;26:1444. doi: 10.1186/s12913-026-15201-x (PMC13689074; doi:10.1186/s12913-026-15201-x)
Supplement: Supplementary file 4 [file 12913_2026_15201_MOESM4_ESM.docx]

**Phase Two: Group discussion**

During the co-design workshop, Groups 1-5 were each assigned one of five delivery methods for the maternity-specific feedback tool (paper-based, electronic, mobile application, audio-based (ACASI), and combined approaches). Discussions highlighted the distinct strengths, limitations, and feasibility of each method across healthcare settings in Malawi. A summary of the group discussions is presented in Additional File 13.

**Group 1: Paper-based tools**

Paper-based tools were considered cost-effective, easy to use, and well-suited for environments with limited digital infrastructure. They do not require internet connectivity and support structured data collection that can minimise omissions. Participants noted that these tools are feasible for basic feedback mechanisms, such as suggestion boxes and exit interviews, but require substantial investment in human and material resources, supervision, and storage when implemented at scale or in more complex formats (e.g., community scorecards or verbal autopsies). Challenges included the need for secure physical storage, vulnerability to data loss during disasters, and manual data management, which can delay analysis and compromise accuracy. Implementing these tools is feasible across all levels of healthcare and is usually overseen by hospital ombudsmen or human resources officers, taking around 30 minutes to complete.

**Group 2: Electronic Tools**

Electronic tools were found to streamline data collection through efficient storage, rapid access to information, and minimal spatial requirements. Their feasibility was considered high with moderate resource input. Limitations included the exclusion of illiterate clients, high maintenance and operational costs, risks of equipment theft, and the need for trained personnel. Electronic feedback mechanisms are suitable for all facility levels and can be self-administered, preferably under the guidance of a neutral party such as an ombudsman.

**Group 3: Mobile applications**

Mobile platforms, including SMS, WhatsApp, Facebook, voice calls, and CommCare, were valued for their flexibility, portability, and real-time data access. Their self-administration potential and short feedback duration (10–15 minutes) were also seen as advantages. However, challenges include network instability, high data costs, and limited interaction (e.g., lack of real-time clarification or probing). Moreover, some platforms, like CommCare, are not entirely self-administered and require staff support.

**Group 4: Audio Tools (ACASI)**

ACASI tools were highlighted for their suitability for clients with visual impairments and for ensuring standardised delivery across sites. They reduce printing costs and improve data accuracy through automated input. Despite their potential, these tools are resource-intensive, requiring technical infrastructure, trained staff, and financial support. Limitations include inaccessibility to clients with hearing impairments, limited flexibility for probing questions, and reliance on stable power sources. Implementation across all levels of care is possible, with tools typically requiring 15–20 minutes to complete.

**Group 5: Combined Approaches**

The combined approach integrates paper, electronic, mobile, and audio tools to enhance system resilience and data reliability, ensuring continuity and accommodating variations in infrastructure. It offers flexibility and inclusivity, especially in contexts with intermittent electricity or diverse user needs. However, the approach demands significant logistical coordination, financial investment, and training. Preparatory steps would require planning for community sensitization, tool integration with existing systems (e.g., DHIS2), and health worker capacity building. Feasibility was considered moderate to high, contingent on facility context, and administration would typically be managed by personnel from the Ombudsman’s office. The tool duration may range from 10 to 20 minutes.

**Group 6: Comparative evaluation by experts**

The technical experts in Group 6 critically assessed all five tool types and contributed additional practical recommendations. Paper-based forms were valued for simplicity and broad familiarity among most health personnel, as well as the ease of producing translated or disability-friendly versions (e.g., Braille). However, scalability, manual data handling, and potential timeliness of data analysis were noted as limitations. Electronic tools offer real-time integration with existing platforms, such as HMIS, and facilitate efficient analysis. However, they face barriers including internet connectivity issues, power supply limitations, cybersecurity concerns, and the need for technical expertise. Mobile applications improve flexibility and portability, but digital literacy and data privacy concerns must be addressed. Audio tools (ACASI) mitigate literacy barriers, but require technical support, stable electricity, and carry similar cost burdens. The combined approach was acknowledged for its resilience, but was described as resource-intensive, inheriting the implementation challenges of each format.

**Cross-cutting themes and practical implementation considerations**

Several cross-cutting themes and practical implementation considerations emerged across all stakeholder groups as essential to the successful rollout and integration of the maternity feedback tool within Malawi’s health system. These include the need for robust community awareness and client sensitisation, sustained stakeholder engagement, targeted capacity building for implementers, and comprehensive sustainability planning, including resource mobilisation. Key enablers for implementation identified across all tool types include translation into local languages, assurance of confidentiality and data protection, fostering client and community acceptance, and securing sufficient logistical and financial support for piloting and scale-up across diverse regions. Building on this, Group 6 provided specific, actionable recommendations to guide effective implementation. These include upholding privacy, confidentiality, and informed consent throughout data collection; ensuring representative sampling by conducting interviews across weekdays, weekends, and public holidays; and selecting pilot districts based on resource availability, facility workload, and regional diversity. Group 6 further recommended assigning dedicated, trained data collectors to minimise disruption to routine care, translating the tool into all widely spoken local languages, and implementing secure data management protocols, such as storing data in locked cabinets within restricted-access areas. To build trust and ensure ethical compliance, clients and stakeholders must be reassured that their information will be handled with the highest standards of privacy and integrity. Finally, convening a dissemination meeting to share pilot findings and planning for potential adaptation of the tool for use in community-based and outreach settings was identified as a key strategy for supporting future scale-up.

**Prototyping, Gallery Walk, and Selection of Tool Delivery Mode**

To support further refinement and stakeholder consensus, a gallery exercise was conducted (Figure 4). This interactive process allowed all participant groups to rotate through stations where the components of the feedback tool were visually presented and analysed. Participants engaged with each tool module, reviewed the findings from other groups, and contributed additional suggestions (see Table 3). The exercise fostered deeper reflection, encouraged collaborative input, and served as a preparatory step for the subsequent voting process on preferred delivery modalities. Following the gallery walk and group presentations, stakeholders participated in a voting exercise to select the most appropriate feedback tool modality for pilot implementation (Figure 5). A total of 51 votes were cast, yielding the results shown in Table 2.

While the electronic tool received the highest number of individual votes, participants reached a consensus to pilot both the paper-based and electronic modalities. The paper-based tool was selected for its accessibility and ease of use across all levels of the health system, particularly in low-resource settings. The electronic version was favoured for its potential to facilitate real-time data collection, analysis, and management. This dual-modality approach was endorsed as a practical and inclusive strategy for the pilot phase, ensuring both feasibility and future scalability of the feedback system.

Following three months of collaboration with the tool designer and two structured rounds of stakeholder feedback, the content of the maternity feedback tool was finalised and compiled into a paper-based format. The tool was systematically organised into three distinct modules, each addressing a specific stage of the maternity care continuum: antenatal care, labour and delivery, and postnatal care. This modular structure was designed to support focused, context-specific, and comprehensive data collection that aligns with each phase of the maternal health service delivery. Each form collected facility-level information and included 24–25 questions covering reception, rapport-building, service provision, communication quality, respectful care, informal payments, disability inclusion, and overall satisfaction. The antenatal form focused on services received during clinic visits; the labour form addressed delivery processes and immediate postpartum care; and the postnatal form assessed care provided to both mother and newborn at 48 hours, 7 days, and 6 weeks. All forms were developed in English and translated into Chichewa and were prepared in both paper-based and electronic formats for validation.
