# Supplementary material for: Co-development of a contextually relevant maternity service user feedback tool in Malawi using participatory consensus methods
Source: BMC Health Serv Res. 2026 Jul 24;26:1444. doi: 10.1186/s12913-026-15201-x (PMC13689074; doi:10.1186/s12913-026-15201-x)
Supplement: Supplementary file 5 [file 12913_2026_15201_MOESM5_ESM.docx]

**Supplementary Table S1: Stakeholder participation**

| **Institution/**  **Organisation type** | **Organisation name** | **No. Invited Co-design** | **No. attended Co-design** | **No. Invited Validation** | **No. attended Validation** |
| --- | --- | --- | --- | --- | --- |
| Ministry of Health and government divisions | Reproductive Health Directorate (RHD) | 5 | 5 | 3 | 3 |
|  | Quality Management Directorate (QMD) and Zonal leaders | 6 | 2 | 6 | 5 |
|  | Digital Health Division (DHD) | - | - | 1 | 0 |
|  | Health Information Systems Program (HISP) | - | - | 1 | 1 |
|  | Clinical Services Directorate | - | - | 1 | 0 |
|  | Nursing and Midwifery Directorate | - | - | 1 | 1 |
|  | **Total** | **11** | **7** | **13** | **10** |
| Training Institutions | Kamuzu University of Health Sciences (KUHeS), Obstetrics and Gynecology department | 1 |  | 1 | 0 |
|  | KUHeS (Midwifery department) | 1 | 1 | 1 | 1 |
|  | Christian Health Association of Malawi (CHAM)Training Institution-Deayang Luke | 1 | 1 | 1 | 1 |
|  | Catholic University of Malawi | - | - | 1 | 1 |
|  | Malawi College of Health Sciences | 1 | 1 | 1 | 1 |
|  | Mzuzu University of Malawi | - | - | 1 | 0 |
|  | **Total** | **4** | **3** | **7** | **4** |
| Professional Associations/  Organizations | Association of Malawi Midwives (AMAMI) | - | - | 1 | 0 |
|  | Association of Obstetrics and Gynecology Doctors in Malawi | - | - | 1 | 1 |
|  | Association of Obstetric Clinical Officers (AOCO) | - | - | 1 | 0 |
|  | **Total** | **0** | **0** | **3** | **1** |
| Community Reps | Maternal Health/Women Groups from communities | 5 | 5 | 5 | 5 |
|  | **Total** | **5** | **5** | **5** | **5** |
| Clinical Services | CHAM Health Services/Hospital | 1 | 1 | 1 | 1 |
|  | Midwives from rural/urban health centres, secondary and tertiary level facilities | 6 | 6 | 6 | 6 |
|  | Clinical officers from primary, secondary level facilities | 3 | 3 | - | - |
|  | Obstetricians from tertiary facility | 2 | 2 | - | - |
|  | District Nursing and Midwifery Officers (managers) | 2 | 2 | - | - |
|  | District Medical Officers (Managers) | 2 | 2 | - | - |
|  | Safe Motherhood Coordinators | 2 | 2 | - | - |
|  | Hospital Ombudsman | 1 | 1 | 3 | 3 |
|  | **Total** | **19** | **19** | **10** | **10** |
| Local NGOs | White Ribbon Alliance (WRA) | 1 | 1 | - | - |
|  | **Total** | **1** | **1** | **-** | **-** |
| Organizations of People with Disability | (Malawi Council for Disability Affairs (MOCODA_ | 1 | 0 | 1 | 1 |
|  | Malawi Union of Blind (MUB) | 1 | 1 | 1 | 1 |
|  | **Total** | **2** | **1** | **2** | **2** |
| Key Donor/international partners | UNICEF | - | - | 1 | 0 |
|  | WHO | 1 | 0 | 1 | 0 |
|  | UNFPA | 1 | 0 | 1 | 0 |
|  | GIZ | 1 | 0 | - | - |
|  | USAID (Momentum 1 and 2-AMREF) | 2 | 1 | 1 | 0 |
|  | PIH | 1 | 0 | - | - |
|  | **Total** | **6** | **1** | **4** | **0** |
| Research institution/Team | Malawi Liverpool Wellcome Research Programme (MLW) | 5 | 5 | 5 | 5 |
|  | Malawi Epidemiology & Intervention Research Unit (MEIRU | 1 | 1 | 1 | 1 |
|  | KUHES | 3 | 3 | 3 | 1 |
|  | Science Communication-Media-MLW | 1 | 1 |  |  |
|  | **Total** | **10** | **10** | **9** | **7** |
| **Grand Total** |  | **58** | **48 (83%)** | **53** | **39(74%)** |
