# Supplementary material for: Co-development of a contextually relevant maternity service user feedback tool in Malawi using participatory consensus methods
Source: BMC Health Serv Res. 2026 Jul 24;26:1444. doi: 10.1186/s12913-026-15201-x (PMC13689074; doi:10.1186/s12913-026-15201-x)
Supplement: Supplementary file 6 [file 12913_2026_15201_MOESM6_ESM.docx]

| **Item** | **ANC** | **Labour and Delivery** | **PNC** | **Other** |
| --- | --- | --- | --- | --- |
| Establishing a Rapport | Did the Healthcare provider greet you? | Did the Healthcare provider greet you? | Did the Healthcare provider greet you? |  |
|  | Did the Health care provider offer you a seat? | Were they friendly, polite and understanding | Were they friendly, polite and understanding | What area as a hospital can improve |
|  | Did the Health care provider introduce her/him-self? | Did the Health care provider introduce her/him-self? | Did the Health care provider introduce her/him-self and were you introduced the PN staff |  |
|  | Were you oriented to the facility | Orientation to labour ward | Orientation to postnatal ward |  |
|  | Did you feel welcomed | Did you feel welcomed | Did you feel welcomed |  |
|  |  | Were you called by your name | Were you called by your name |  |
|  |  | Did they use the language that you can understand | Did they use the language that you can understand |  |
| Payment | Did you pay for any services | Did you pay for any services | Did you pay for any services |  |
|  |  |  |  |  |
| Health education/information giving and communication | Did the Provider offer you health education?  Were you able to understand | Refer Education component for Labour and Delivery | Refer postnatal matrix (family planning, care after negative outcomes/causes for loss, etc |  |
|  | Were you given instruction how to use ITNs | Did the provider continue to update you on the progress of labour | Were you communicated the reason for caesarean section |  |
|  | What kind of information have you received | Did the HCW effectively communicate | Were you educated on cord care, breastfeed position and attachment, C/S incision care,Lochia changes |  |
|  | Did they give you information about what to expect during ANC | Did you discuss the processes which may be required during labour and delivery/emergencies | Did they explain to you about the postnatal visits ie 1 week and 6 weeks visits |  |
|  | Did HCW take his/her time to explain care to be administered /referral and their rationale | Were you communicated the type of care and treatment you received | Did HCW take his/her time to explain care to be administered /referral and their rationale |  |
|  | Did HCW explained the results of the findings after assessment | Did HCW explained the results of the findings after assessment | Did HCW explained the results of the findings after assessment |  |
|  | Were you able to understand | Were you told the condition of the baby | Were you explained to on the outcome of the baby, condition and sex of the baby |  |
|  | Did provider review your knowledge on ANC | Were you told the time of delivery of your baby |  |  |
|  | What type of information did you receive regarding birth preparedness (danger signs) Refer to ANC matrix | What type of information did you receive regarding birth preparedness (danger signs) |  |  |
|  | Did HCW give you enough time to verbalise your concerns |  |  |  |
|  | Did you have a chance to ask questions | Did you have a chance to ask questions | Did you have a chance to ask questions |  |
|  | Did the HCW answer your question satisfactory | Did the HCW answer your question satisfactory | Did the HCW answer your question satisfactory |  |
| Services received | What services did you receive (refer to ANC matrices) | What services did you receive during labour and delivery (Frequency of examinations, skin to skin, delivery positions etc, refer labour and delivery processes, complications, 3^rd^ stage delivery) | Did they register your child for birth registration |  |
|  |  |  | What services did you receive during postnatal (Frequency of examinations during hospitalisation/before discharge, breastfeeding, VE inspection and abdominal exam etc.  Baby examinations,  Immediate postnatal care |  |
|  | Were you assisted by skilled health care worker? | Were you assisted by skilled health care worker? | Were you assisted with mental health assessment and care |  |
|  |  | Were you given any pain relief during labour/ tear suturing | Were you given any pain relief after caesarean section |  |
|  |  |  | Were you nursed on a bed or mattress only and were you cleaned and left dry after delivery |  |
|  |  |  | Did the baby received vaccinations before discharge |  |
| Satisfaction | Do you think you would come to this facility gain? | Do you think you would come to this facility gain? | Do you think you would come to this facility gain? |  |
|  | Would you recommend a friend or relative to seek ANC at this facility? | Would you recommend a friend or relative to seek labour and delivery services | Would you recommend a friend or relative to seek postnatal services |  |
|  | Do you feel the services were comprehensive | Were your concerns addressed |  |  |
|  | What services were you satisfied with/like | What services were you satisfied with/like | What services were you satisfied with/like |  |
|  |  | Was the delivery beds user friendly? |  |  |
|  | What services were you not satisfied with/dislike | What services were you not satisfied with/dislike | What services were you not satisfied with/dislike |  |
|  | What services did you expect | What services did you expect | What services did you expect |  |
|  | Are you satisfied with the overall ANC services | Were you happy with how the labour ward teams interacted with each other |  |  |
|  | How could you rate the ANC services received out of 10 | How could you rate the labour and delivery services received out of 10 | How could you rate the postnatal services received out of 10 |  |
|  |  |  |  |  |
| Privacy and confidentiality | Was there privacy and confidentiality during care provision | Was there privacy and confidentiality during care provision | Was there privacy and confidentiality during care provision |  |
| Respect and rights (RMC)  Companionship | Did you feel respected during ANC care | Was there any form of physical and sexual abuse | Were your religious beliefs respected |  |
|  | Did you feel that your rights were respected during ANC care | Were you allowed to have a guardian/did you feel supported during labour and delivery | Did you feel respected during PNC care |  |
|  | Were you treated with respect and dignity | Did you feel that your rights were respected during labour and delivery | Did you feel that your rights were respected during PNC care |  |
|  |  |  | Were you treated with respect and dignity |  |
|  |  |  | Was there any form of physical and sexual abuse |  |
|  |  |  | Were you forced to stay in the hospital against your will |  |
| Areas of improvement | What should improve in ANC services | What needs to be done during labour and delivery to improve services | What needs to improve at postnatal | What should the facility do to serve you better |
| Number of visits | How many visits have you attended |  |  |  |
| Areas of care | Initial/subsequent/  Maternity waiting home/ward |  |  |  |
|  | Have you given you a date for the next visit? |  |  |  |
| Establishing a Rapport |  | Were they friendly or understanding in labour ward |  |  |
| Waiting time | How long did it take to get helped? | How long did it take for you to be attended to (various stages e.g. latent, second and third stage of labour) |  |  |
|  | How long have you waited to be assisted |  |  |  |
|  | What time did you arrive |  |  |  |
|  | How satisfied were you with the waiting time |  | How many times have you been attended to since you gave birth |  |
| HCW attitude | What was HCW attitude? /Friendly | What was HCW attitude? /Friendly | What was HCW attitude? /Friendly |  |
| Sanitation and environment | How satisfied were you with sanitation | How satisfied were you with sanitation | How satisfied were you with sanitation |  |
|  |  |  | Were the facilities allowing mother and baby to be together 24/7 |  |
| Disability friendly services | Was the facility disability friendly |  |  |  |
| Consent |  | Was consent sought before procedures e.g. Caesarean section |  |  |
| Ombudsman office |  | Do you know that there is an office of Ombudsman office to lodge complaints? | Do you know that there is an office of Ombudsman office to lodge complaints? | Were you informed where to lodge complaints |
|  |  |  | Were your complaints attended to |  |
| If applicable bereavement care |  |  | If applicable, did you feel supported after the loss/empathetic |  |
| Follow up care | Were you informed on next appointment date |  | Were you informed on next appointment date |  |
| Heath Education |  | Did the health care provider speak to you in a polite manner | Did the provider give health education on breastfeeding |  |
|  |  | Did they teach you how to take care of a new-born? |  |  |
|  |  | Did the provider gave you information on any complications |  |  |
| Waiting time | How long did it take to get helped? | How long did it take for you to be attended to? |  |  |
|  | How long have you waited to be assisted |  |  |  |
|  | What time did you arrive |  |  |  |
|  | How satisfied were you with the waiting time |  | How many times have you been attended to since you gave birth |  |
|  | Did the health care provider gave you time to voice out your concerns? | Was privacy/confidentiality provided throughout the time of labour and delivery | Did the health care provider explain to you the immediate postnatal care for the baby |  |
|  | Did the provider examine you and give you feedback? | Did the Health care worker asked you to consent to be examined? | Was privacy/confidentiality provided throughout the time after delivery? |  |
|  | How satisfied were you with the provider’s professionalism | Did the Health care provider give you time to ask questions | Did they explain what to expect after delivery |  |
|  | Did the provider review your knowledge on ANC? | Did the Healthcare provider explain the process of labour and delivery and what is expected to do | Did they explain how to take care of the baby and were you able to understand |  |
|  |  | Did the provider use language you can understand | Did the provider explain to you for postnatal visits? |  |
|  |  | Was respectful maternity care provided i.e privacy |  |  |
|  |  | How did you find the cleanliness of the facility |  |  |
|  |  | Were you delivered by a skilled birth attendant? |  |  |
|  |  | Are you satisfied with the overall care? |  |  |
| Health centre | Did the Health care provider explain to you why you are being referred | Did the Health care provider explain to you why you are being referred |  |  |
| District & Central Hospital | Did the Healthcare worker explained to you why you have been referred | Did the Healthcare worker explained to you why you have been referred |  |  |
