# Supplementary material for: Co-development of a contextually relevant maternity service user feedback tool in Malawi using participatory consensus methods
Source: BMC Health Serv Res. 2026 Jul 24;26:1444. doi: 10.1186/s12913-026-15201-x (PMC13689074; doi:10.1186/s12913-026-15201-x)
Supplement: Supplementary file 7 [file 12913_2026_15201_MOESM7_ESM.docx]

| **Group** | **Tool** | **Advantages** | **Disadvantages** | **Feasibility** | **Steps/needs before implementing** | **Which facilities and by who** | **Length of the tool** |
| --- | --- | --- | --- | --- | --- | --- | --- |
| 1 | Paper based | Do not require internet/network | Requires more time to complete | Minimal support with exit interviews and referrals, suggestion box and may need full support with training if verbal autopsy tool, community score card and participatory referral appraisal | Human and material (stationary, infrastructure, transport) resources, training, supervision review meetings | All facility levels depending on the tool used. It can be implemented by hospital ombudsman and HR | Not more than 30 minutes |
|  |  | Cheap/cost effective | Storage space |  |  |  |  |
|  |  | Structured so that provider cannot miss components | Dat management issues |  |  |  |  |
|  |  | Easy to use | Can not be retrieved if natural disasters |  |  |  |  |
|  |  |  | Suggestion box not friendly for illiterate people |  |  |  |  |
|  |  |  | Costly printing |  |  |  |  |
| 2 | Electronic tool | Easy to handle | Not for illiterates | Feasible with minimal support-resources, time and information updates |  | All facility levels and self-administered with neutral person (ombudsman) |  |
|  |  | Easy storage | Expensive operating cost |  |  |  |  |
|  |  | No need for a special room | High Maintenance cost |  |  |  |  |
|  |  | Data safety | Training of persons |  |  |  |  |
|  |  | Fast way to get information | Theft of gadgets |  |  |  |  |
|  |  | firsthand information |  |  |  |  |  |
|  |  |  |  |  |  |  |  |
| 3 | Mobile App | Has more delivery methods like WhatsApp, SMS, Facebook, Audio call, Comcare app | Network challenges and data bundles | Depends on tool used minimal support for other tools and so much support for others |  |  | 10-15 minutes |
|  |  | Data access in real time | Inconvenient if required in time |  |  |  |  |
|  |  | self-administered | No opportunity for clarification |  |  |  |  |
|  |  | Flexibility | Costly/not sustainable |  |  |  |  |
|  |  | Audio call disability friendly | Need uploading the app |  |  |  |  |
|  |  |  | Comcare not self-administered |  |  |  |  |
| 4 | Audio tools ACASI | Can be beneficial to those with visual impairment | Those with hearing /visual impairment cannot get the message | It’s feasible with huge support- due to cost of maintenance, funding challenges, attitude of staff, fear from community members, expensive as needs gadgets, capacity building of staff | Needs question formulation, purchasing the gadgets, uploads the questions, tool validation identifies the sites and test, modify the tool and roll out implementation | All levels of care | 15-20 minutes long |
|  |  | Consistency across all the sites where we are implementing the tool | Maintenance (sustainability) |  |  |  |  |
|  |  | Saves resources, cost of printing and papers | Requires skilled personnels in ICT |  |  |  |  |
|  |  | Storage of data is easy | If not properly secured, data can be tampered or lost |  |  |  |  |
|  |  | Cost effective in relation to employing data collectors | Lost data cannot be easily retrieved |  |  |  |  |
|  |  |  | Increases unemployment rate |  |  |  |  |
|  |  |  | Hard to probe more information since its pre recorded |  |  |  |  |
|  |  |  | Requires more time to complete questionnaire |  |  |  |  |
|  |  |  | Needs power, electricity and solar |  |  |  |  |
| 5 | Combined approaches | Minimize loss of data if one system fails | Biased on tool that is easy to use | Feasible but will require so much support due to gadgets, internet, recorder, papers, printing costs | Community awareness | It depends on facility setup and situations and can be administered by Ombudsman office | 10-20 minutes |
|  |  | Flexibility | Needs more resources |  | HCWs awareness |  |  |
|  |  | No service interruptions if resource constraint | Capacity building |  | Clients sensitisation |  |  |
|  |  | Equal access to the services even with no electricity |  |  | lobby inclusion in DHSI2 |  |  |
|  |  | Capacity building exposure to different approaches |  |  | Review existing tools |  |  |
|  |  |  |  |  | Sustainability plan with government |  |  |
