# Supplementary material for: Co-development of a contextually relevant maternity service user feedback tool in Malawi using participatory consensus methods
Source: BMC Health Serv Res. 2026 Jul 24;26:1444. doi: 10.1186/s12913-026-15201-x (PMC13689074; doi:10.1186/s12913-026-15201-x)
Supplement: Supplementary file 8 [file 12913_2026_15201_MOESM8_ESM.docx]

| **Question/information/section** | **ANC form** | **Labour and delivery form** | **Postnatal form** |
| --- | --- | --- | --- |
| Title of the form |  | Rephrased in both languages |  |
| Facility information | Agreed to be completed by facility staff or ombudsman | Edited on location to be postnatal and labour wards | Edited on location in Chichewa version |
|  | Changes to responses on facility ownership | Same changes as ANC form | Same changes as ANC |
|  | Made changes in Chichewa version | Same changes as ANC form | Same changes as ANC |
| Demographic data | Added age, level of education, disability information and type of disability if services accommodate the disability | Similar changes as ANC form and to add condition of the baby | Similar changes as labour ward form but added date of delivery and mode of delivery |
| Reception and rapport building | Rephrased two questions | Same changes as ANC form | Same changes as ANC form |
| Services received | Changed medical to non-medical terms on history taking, physical exam, obstetric exam and laboratory investigations both English and Chichewa version | Rephrased a question related to vaginal examination, contractions and initiation of labour both languages and added who conducted the delivery | Rephrased questions on anal and vaginal care, separated care of mother and baby. For those who lost babies added mental health support care, counselling, family planning and information on causes of loss |
| Waiting time | Rephrased the question to ask satisfaction during initial and subsequent visit | Rephrased to ask satisfaction with waiting time during admission to labour ward | Rephrased to ask satisfaction with waiting time during postnatal care |
| Respectful Maternity Care |  | Rephrased on companionship |  |
| Form of abuse | Rephrased the questions in both languages |  |  |
| Communication and provider interaction | Rephrased the questions in both languages | add if informed time of delivery and sex of the baby | If informed reason of baby admission to nursery and kangaroo if applicable and HIV exposed baby care |
| Disability information | Moved to follow demographics | Similar changes as ANC form and to add condition of the baby |  |
| Payment | If yes, need to explain | If yes, need to explain | If yes, need to explain |
| Clients’ expectations | Rephrased in both languages | Rephrased in both languages | Rephrased in both languages |
| Client satisfaction | Rephrased in both languages | Rephrased in both languages | Rephrased in both languages |
