# Supplementary material for: Co-development of a contextually relevant maternity service user feedback tool in Malawi using participatory consensus methods
Source: BMC Health Serv Res. 2026 Jul 24;26:1444. doi: 10.1186/s12913-026-15201-x (PMC13689074; doi:10.1186/s12913-026-15201-x)
Supplement: Supplementary file 9 [file 12913_2026_15201_MOESM1_ESM.docx]

**Supplementary Figure S1: Development pathway of the maternity-specific feedback tool**
